# Supplementary figures and images for: Metabolomic and physiological analysis of bud differentiation in dense apple (Malus×domestica Borkh.) orchards following thinning and reshaping
Source: PeerJ. 2025 Sep 30;13:e20011. doi: 10.7717/peerj.20011 (PMC12493702; doi:10.7717/peerj.20011)

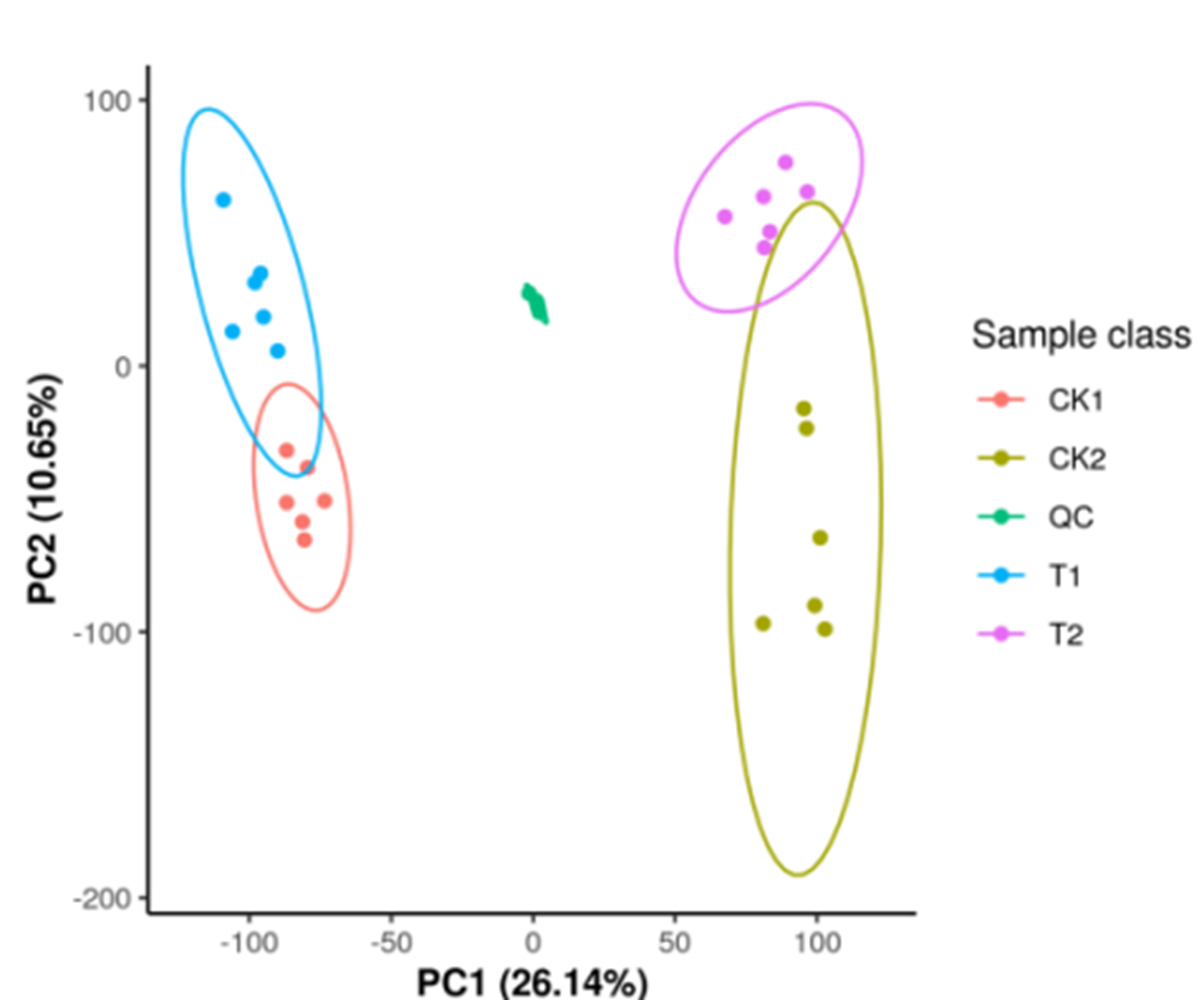

Supplement: Supplemental Information 4 [file peerj-13-20011-s004.png]
